# Illumina sequencing-based community analysis of bacteria associated with different bryophytes collected from Tibet, China

**DOI:** 10.1186/s12866-016-0892-3

**Published:** 2016-11-16

**Authors:** Jing Yan Tang, Jing Ma, Xue Dong Li, Yan Hong Li

**Affiliations:** College of Life Science, Capital Normal University, Xisanhuan North Road 105#, Haidian District, Beijing, 100048 China

**Keywords:** Illumina-sequencing, Bacterial diversity, Community, Bryophytes, Phylogeny status

## Abstract

**Background:**

Previous studies on the bacteria associated with the bryophytes showed that there were abundant bacteria inhabited in/on these hosts. However, the type of bacteria and whether these discriminate between different bryophytes based on a particular factor remains largely unknown.

**Results:**

This study was designed to analyze the biodiversity and community of the bacteria associated with ten liverworts and ten mosses using Illumina-sequencing techniques based on bacterial 16S rRNA gene. A total of 125,762 high quality sequences and 437 OTUs were obtained from twenty bryophytes. Generally, there were no obvious differences between the richness of bacteria associated with liverworts and mosses; however, the diversity was significantly higher in liverworts than in mosses. The taxonomic analyses showed that there were abundant bacteria inhabited with each bryophyte and those primarily detected in all samples were within the phyla *Proteobacteria*, *Actinobacteria*, *Acidobacteria*, *Bacteroidetes*, *Armatimonadetes* and *Planctomycetes*. In addition, bacteria assigned to *Chloroflexi, Fibrobacteres*, *Gemmatimonadetes*, *Chlamydiae*, group of TM6 and WCHB1-60 also appeared in part of the bryophytes. The assigned bacteria included those adapted to aquatic, anaerobic and even extreme drought environments, which is consistent with the bryophyte transition from aquatic to terrestrial conditions. Of them, approximately 10 recognized genera were shared by all the samples in a higher proportion, such as *Burkholderia*, *Novosphingobium*, *Mucilaginibacter*, *Sorangium*, *Frankia*, *Frondihatitans*, *Haliangium*, *Rhizobacter*, *Granulicella* and *Hafnia,* and 11 unclassified genera were also detected in all samples, which exhibited that large amounts of unclassified bacteria could interact with the bryophytes. The Heatmap and Principle Coordinate Analyses showed that bacteria associated with six mosses displayed a higher community similarity. Notably, the bacteria associated with another four mosses exhibited higher similarity with the ten liverworts.

**Conclusions:**

The result of further analysis of the bacterial community in different bryophytes revealed that the phylogeny of hosts might portray a strong influence on the associated bacterial community and that niche also played important roles when the hosts were phylogenetically more similar. Further studies are needed to confirm the role of phylogeny on bacterial communities and determine the level of influence on predicting which bacteria is associated with the host.

## Background

The presence of microbes is essential to the overall health of all plants in our environment [1]. Many of the bacteria associated with host plants work to degrade organic pollutants, improve plant growth [2–4] or enhance hosts the capability of adapting extreme environment [5]. Of the nearly all the plant species that exist on the earth, each individual plant is host to many bacteria [6]. Despite this widespread dependence, the majority of related research has concentrated on only the common higher plants and few reports have incorporated effects on community structure or the diversity of bacteria associated with plants such as bryophytes [7–9].

Bryophytes are the simplest land plants and form the basal clade of land plants. They are considered to be the ancestors of pteridophytes and all other tracheophytes. They represent the first green plants to colonize the terrestrial environment [10], and have evolved numerous important adaptations, including the alternation of gametophytic and sporophytic generations, elaboration of gametophytes, specialization of gametangia, and the adaption of desiccation-resistant spore walls [11]. Thus, bryophytes adapt to various environments ranging from harsh Antarctic conditions to extremely drought niches by their ability to preserve both water and many nutrients in unfavorable environments [12]. Importantly, bryophytes play important roles in nutrient cycling and can act as bio-indicators of air pollutants or heavy metals, making them crucial to the environmental health of many ecosystems [11].

A recent study of *Grimmia montana* [13] and other bryophytes [8, 14, 15] showed that there were abundant bacteria associated with these hosts. However, the type of bacteria that inhabits in/on bryophytes and whether these discriminate between different bryophytes based on a particular factor remains largely unknown. Thus, we studied 20 different types of bryophyte samples, including ten liverworts and ten mosses collected from Tibet, China, in order to determine their associated bacteria based on the Illumina-sequencing of 16S rRNA gene amplicons. The aims of this study were to find out which bacteria were dominant in/on the different bryophytes, what was the differences between the bacteria associated with the bryophytes and common higher plants, and which factor made a strong influence on the bacteria distribution in/on these bryophytes. The results of this study would be helpful to improve our understanding of the composition of bacteria associated with these hosts, and also could provide valuable insight into the mechanism of interactions between microbes and bryophytes.

## Methods

### Plant material and surface treatment

The investigation concentrated on the biodiversity and community structure of bacteria associated with 20 kinds of bryophytes, which were collected from four sites (shown in Table 1) in Tibet, China at Oct 13-15th, 2014. These bryophytes were not in the list of national key protected wild plants (http://guoqing.china.com.cn/2012-11/01/content_26977329.htm) and allowed to free collection for this survey (http://rep.iplant.cn/news/27). After the bryophytes were sampled, they were stored at 4 °C and transferred into the lab as soon as possible. In total, the samples included 10 types of liverworts and 10 types of mosses, which were labeled by _T or _X, respectively (shown in Table 1). Samples were first cleaned by rinsing several times with tap water to remove the attached matrix, and then washed by sterilized water for three times. Subsequently, the samples were surface treated by 70% ethanol for 3 min, which was followed by washing five times with sterile distilled water. Finally, the water was absorbed by the sterilized filter paper and the surface treated samples were placed into the sterile glass dish for use.

**Table 1:** Information of all samples

Sample ID^a^	Taxonomic status	Scientific Name	Locality	Longitude/Latitude	Altitude (m)
JM1_T	Jungermanniales Plagiochilaceae	*Plagiochilion mayebarae* S. Hatt.	Zhamo roadside	N29° 48′ 07.17″ E95° 41′ 58.70″	4100
JM2_T	Jungermanniales Lepidoziaceae	*Lepidozia reptans* (L.) Dum.	Zhamo roadside	N29° 48′ 07.17″ E95° 41′ 58.70″	4100
JM28_T	Jungermanniales Jungermanniaceae	*Jamesoniella elongella* (Tayl.) Steph.	Zhamo roadside	N29° 45′ 16.57″ E95° 42′ 38.39″	4175
JM30_T	Jungermanniales Jungermanniaceae	*Jungermannia parviperiantha* Gao et Bai	Zhamo roadside	N29° 45′ 16.57″ E95° 42′ 38.39″	4175
GL3_T	Jungermanniales Jungermanniaceae	*Jungermannia leiantha* Grolle.	Galongla glacier	N29° 48′ 07.17″ E95° 41′ 58.70″	3993
GL7_T	Jungermanniales Jungermanniaceae	*Jungermannia leiantha* Grolle.	Galongla glacier	N29° 48′ 07.17″ E95° 41′ 58.70″	3993
GL9_T	Marchantiales Gymnomitriaceae	*Apomarsupella revolute* (Nees) Schust.	Galongla glacier	N29° 48′ 07.17″ E95° 41′ 58.70″	3993
GL10_T	Jungermanniales Jungermanniaceae	*Jungermannia parviperiantha* Gao et Bai	Galongla glacier	N29° 48′ 07.17″ E95° 41′ 58.70″	3993
GWL5_T	Jungermanniales Jungermanniaceae	*Horikawaella rotundifolia* Gao et Yi	Gawalong glacier	N29° 45′ 10.75″ E95° 42′ 23.61″	4337
GWL7_T	Marchantiales Gymnomitriaceae	*Apomarsupella revolute* (Nees) Schust.	Gawalong glacier	N29° 45′ 10.75″ E95° 42′ 23.61″	4337
JM3_X	Polytrichales Polytrichaceae	*Pogonatum urnigerum* (Hedw.) Beauv.	Zhamo roadside	N29° 48′ 07.17″ E95° 41′ 58.70″	4100
JM5_X	Hypnales Hypnaceae	*Hypnum revolutum* (Mitt.) Lindb.	Zhamo roadside	N29° 48′ 07.17″ E95° 41′ 58.70″	4100
JM25_X	Hypnales Amblystegiaceae	*Sanionia uncinata* (Hedw.) Loeske	Zhamo roadside	N29° 45′ 16.57″ E95° 42′ 38.39″	4175
JM29_X	Grimmiales Grimmiaceae	*Racomitrium himalayanum* (Mitt.) Jaeg.	Zhamo roadside	N29° 45′ 16.57″ E95° 42′ 38.39″	4175
TM2_X	Hypnales Hypnaceae	*Hypnum plumaeforme* Wils.	Tongmai Roadside	N30° 3'46.56" E95° 9'35.45"	2047
GL1_X	Grimmiales Grimmiaceae	*Racomitrium barbuloides* Card.	Galongla glacier	N29° 48′ 07.17″ E95° 41′ 58.70″	3993
GL4_X	Polytrichales Polytrichaceae	*Pogonatum perichaetiale* (Mont.) Jaeg.	Galongla glacier	N29° 48′ 07.17″ E95° 41′ 58.70″	3993
GL5_X	Grimmiales Grimmiaceae	*Racomitrium sudeticum* (Funck.) Bruch. et Schimp.	Gawalong glacier	N29° 45′ 10.75″ E95° 42′ 23.61″	4337
GWL2_X	Hypnales SematopHyllaceae	*Wijkia deflexifolia* Crum	Galongla glacier	N29° 48′ 07.17″ E95° 41′ 58.70″	3993
GWL9_X	Hypnales Brachytheciaceae	*Brachythecium rutabulum* (Hedw.) B. S. G.	Gawalong glacier	N29° 45′ 10.75″ E95° 42′ 23.61″	4337

^a^T stands for liverwort and X stands for moss

### DNA extraction and PCR amplification of the bacterial 16S rRNA gene

Approximately 1 g (including about 50 ~ 200 individuals) of each bryophyte sample was homogenized in liquid nitrogen, and then total DNA was extracted using the FastDNA SPIN Kit for Soil (MPBio) according to the instructions provided by the manufacturer. The first 16S rRNA gene amplification of bacteria associated with 20 bryophytes was conducted by using the Taq Mix kit (2 × Taq PCR Master Mix, Biomed) in a total volume of 50 μl containing 25 μl Taq Mix and 20 μl ddH_2_O, 2 μl of each primer, and 1 μl template DNA based on the primers 799 F (5′-AACAGGATTAGATACCCTG-3′) and 1492R (5′-GGTTACCTTGTTACGACTT-3′) [16]. The thermal cycling conditions were as follows: an initial denaturation at 94 °Cfor 3 min, then 30 cycles of 94 °C for 30 s, 55 °C for 30 s, and 72 °C for 1 min, with a final extension step of 72 °C for 5 min. PCR products were separated by the electrophoresis technique using a 1% agarose gel. The bands of approximately 730 bp in size were excised and purified by the TIAN gel Mini Purification Kit (TIAN GEN Co.) as described by the manufacturer.

### Nested-PCR amplification and Illumina sequencing

Purified PCR products of the bacterial 16S rRNA gene were used for a second amplification step based on the primers 926 F (5′-AAACTYAAAKGAATTGACGG-3′) and 1392R (5′-ACGGGCGGTGTGTRC-3′). To distinguish the different samples, a barcoded-tag with eight nucleotide bases was randomly added to the upstream of the universal primers. The second PCR amplification was carried out by using the TransStart Fastpfu DNA Polymerase in a total volume of 20 μl containing 4 μl 5 × FastPfu Buffer, 2 μl ddH_2_O, 2 μl 2.5 mM dNTPs, 0.8 μl Forward Primer (5 μM), 0.8 μl Reverse Primer (5 μM), 0.4 μl FastPfu Polymerase and 10 ng template DNA. The thermal cycling conditions were as follows: an initial denaturation of 95 °C for 3 min, and 27 cycles of 95 °C for 30 s, 55 °C for 30 s and 72 °C for 45 s and a final extension step of 72 °C for 10 min. Amplicons were extracted from 2% agarose gels and purified using the AxyPrep DNA Gel Extraction Kit (Axygen Biosciences, Union City, CA, U.S.) according to the manufacturer’s instructions and quantified using QuantiFluor™ -ST (Promega, U.S.). Purified amplicons were pooled in equimolar concentrations and sequenced using 300 bp paired-end model with the MiSeq system (Illumina, USA) in Majorbio (Shanghai).

### Bioinformatic analysis of the sequences

The lengths of the short reads were extended by identifying the overlap between paired-end reads by the FLASH software [17]. The singleton sequences were removed and the low quality sequences were filtered out using QIIME software (version 1.17) [18]. Reads that were not assembled were also discarded. The reads were sorted according to barcode sequences and the sample sources. The sequences number of each sample was counted. Sequences covered the V6-V8 region of bacterial 16S rRNA gene were clustered into operational taxonomic units (OTUs) at 97% sequence similarity by using UPARSE software (version 7.1 http://drive5.com/uparse/) [19] and chimeric sequences were identified and removed using UCHIME [20].

To further calculate the Alpha diversity, species richness (Chao), species coverage (Coverage), species diversity (Shannon-Wiener Index and Simpson’s diversity index) and rarefaction analyses were calculated using the software of Mothur version v.1.30.1 [21]. The richness index Chao estimator, was used to estimate the richness of the bacteria [22]. Shannon diversity and Simpson indexes were used to estimate the biodiversity of the bacterial communities. These alpha diversity indexes were compared between sample groups using Two Independent Sample tests of nonparametric analysis in SPSS version 16.0 for Windows (SPSS Inc., Chicago, IL). We analyzed the taxonomy of each 16S rRNA gene sequence with RDP Classifier [23] (http://rdp.cme.msu.edu/) against the Silva 16S rRNA database using a confidence threshold of 70% [24]. Community structure analyses were based on the phylum and genus taxonomy levels.

Heatmaps were generated on the basis of the relative abundance of phyla and genera, respectively, using R (version 2.15; The R Project for Statistical Computing, http://www.R-project.org). For phylogeny-based cluster comparisons, the composition of the microbial communities present in the samples of ten liverworts, ten mosses and all these twenty bryophytes were analyzed based on the Bray-Curtis distance and principal coordinate analysis (PCoA) plots were generated.

The two groups of bryophytes (obtained by the analysis of PCoA plots) specific to different bacteria types was performed using the linear discriminant analysis (LDA) effect size (LEfSe) method (http://huttenhower.sph.harvard.edu/lefse/) for biomarker discovery, which emphasizes both statistical significance and biological relevance. With a normalized relative abundance matrix, LEfSe uses the Kruskal-Wallis rank sum test to detect features with significantly different abundances between assigned taxa and performs LDA to estimate the effect size of each feature. A significance alpha of 0.05 and an effect size threshold of 4 were used for all biomarkers discussed in this study. All tests for significance were two-sided, and *p* values below 0.05 were considered statistically significant.

## Results

### Amplicon analyses by illumina sequencing

The statistical results showed that 305,486 sequences were obtained from the 730 bp amplified products derived from the total DNA extracted from twenty bryophytes. After removing the low quality data, the singleton sequences and the chimeric sequences, about 125,762 high quality sequences were obtained. Totally more than ninety-nine percent of these sequences were classified as bacteria and their average length was 479 bp.

### Bacterial species richness and diversity in different bryophytes

**Fig. 1:**
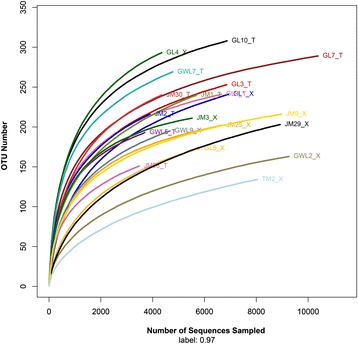
Rarefaction curves of bacterial 16S rDNA gene sequences

The overall number of OTUs detected by the analysis reached 437, based on ≥97% nucleotide sequence identity between sequences. To assess whether our sampling effort provided sufficient OTU coverage of the bacterial composition associated with each bryophyte, rarefaction curves and the library coverage were generated for each sample. The rarefaction curves and >98.5% coverage in all samples showed that the libraries could reflect the main bacterial information in each sample (Fig. 1). Of 437 OTUs detected overall, 387 OTUs could be detected in both liverwort and moss species. Thirty-one special OTUs were only detected in liverworts, while only 19 special OTUs were found in mosses. In addition, the OTU number was varied from 143 (*Hypnum plumaeforme*, TM2_X) to 308 (*Jungermannia parviperiantha*, GL10_T) in different samples. In general, the average OTU number was 240 in liverworts, which was slightly higher than the average OTU number in mosses but was not significantly different (*p* > 0.05). There was no relationship between the OTU numbers and the sampling sites (Table 2).

**Table 2:** The diversity indexes of bacteria associated with all the bryophytes

Sample ID	Reads	OTUs	Chao estimator	Coverage (%)	Shannon diversity	Simpson
JM1_T	5693	240	320 ± 51.5	98.8	3.95 ± 0.04	0.039 ± 0.002
JM2_T	3896	216	267 ± 35.5	98.5	4.15 ± 0.05	0.029 ± 0.002
JM28_T	3475	151	198 ± 39.5	98.7	3.44 ± 0.05	0.07 ± 0.005
JM30_T	4350	240	285 ± 30.5	98.6	3.7 ± 0.06	0.071 ± 0.004
GL3_T	6857	253	310 ± 39.5	99.1	4.1 ± 0.04	0.032 ± 0.001
GL7_T	10382	289	334 ± 32.5	99.5	4.1 ± 0.04	0.041 ± 0.002
GL9_T	6653	241	288 ± 36.5	99.3	4.12 ± 0.04	0.032 ± 0.001
GL10_T	6869	308	357 ± 34.5	99.1	4.58 ± 0.04	0.020 ± 0.001
GWL5_T	3697	193	231 ± 31	98.8	3.95 ± 0.05	0.042 ± 0.003
GWL7_T	4771	269	346 ± 51.5	98.6	4.44 ± 0.04	0.024 ± 0.001
JM3_X	5521	211	233 ± 20.5	99.4	3.95 ± 0.04	0.042 ± 0.002
JM5_X	8966	216	256 ± 31.5	99.5	3.47 ± 0.04	0.083 ± 0.004
JM25_X	6662	202	262 ± 49.5	99.3	3.01 ± 0.06	0.18 ± 0.009
JM29_X	8919	203	270 ± 43.5	99.2	2.22 ± 0.04	0.25 ± 0.008
TM2_X	8035	134	191 ± 42.5	99.3	1.89 ± 0.04	0.27 ± 0.007
GL1_X	6929	241	317 ± 50	99.0	2.81 ± 0.05	0.19 ± 0.008
GL4_X	4349	293	328 ± 24	98.6	4.41 ± 0.05	0.032 ± 0.002
GL5_X	9263	163	207 ± 33	99.4	2.37 ± 0.04	0.20 ± 0.006
GWL2_X	5808	173	214 ± 30	99.1	2.34 ± 0.06	0.23 ± 0.009
GWL9_X	4667	195	250 ± 40.5	98.8	3.57 ± 0.05	0.064 ± 0.004

The Chao estimator showed that the richness of the bacteria associated with these bryophytes ranged from 191 to 357. The average Chao estimator was 294 in liverworts, which was also slightly higher but not significantly (*p* > 0.05) different than that detected in mosses (253). However, the Shannon diversity indexes of bacteria associated with liverworts exhibited a much greater value than those with mosses (*p* < 0.05). The average Shannon diversity index was 4.053 in liverwort samples, whereas it was only 3.004 in mosses. Interestingly, the Shannon diversity indexes were nearly similar between different liverwort samples, but relatively bigger changes among different moss species (Table 2).

### Community structure of bacteria associated with the bryophytes at phylum level

**Fig. 2:**
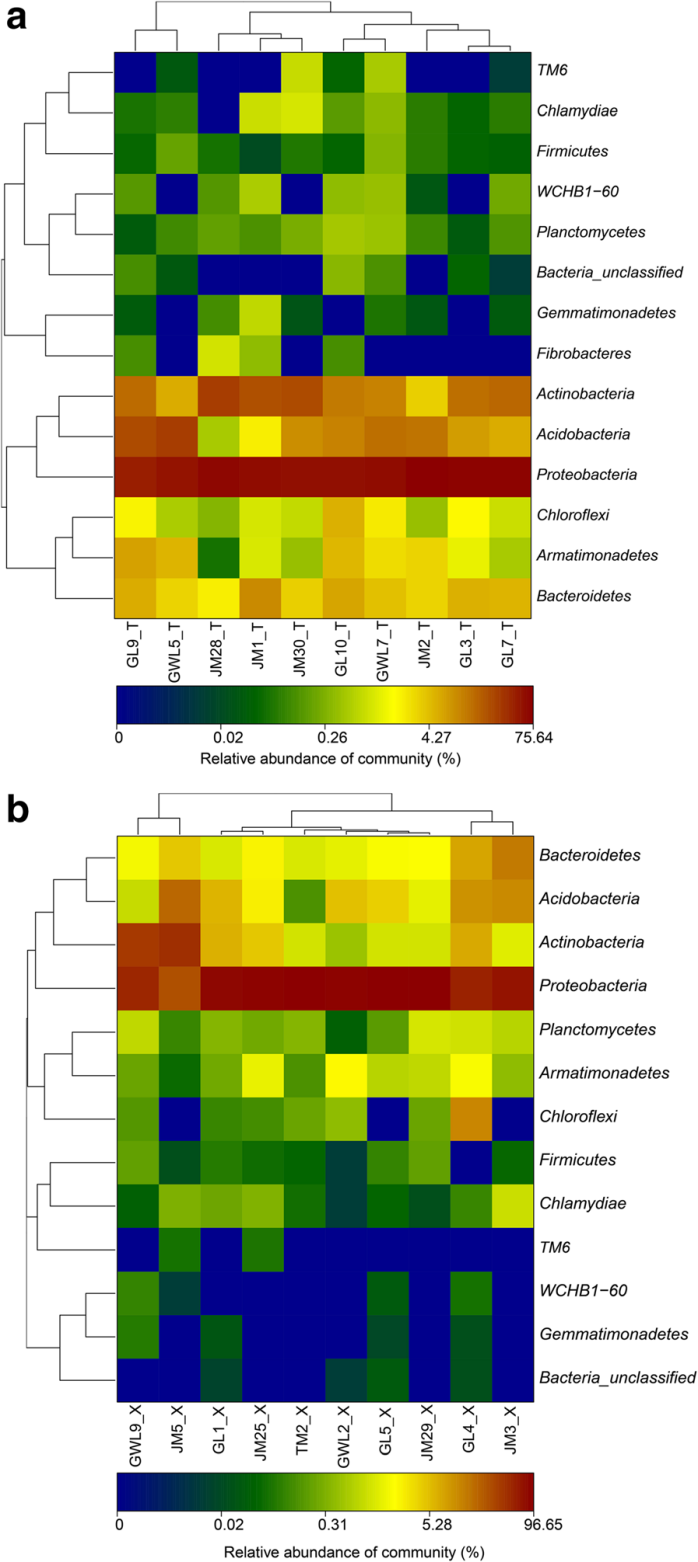
The distribution of all sequences from each bryophyte sample on the phyla (**a** and **b**) level

According to the sequencing results, the phylum of less than 2% OTUs could not be identified (Table 3). For the other 98% of OTUs, bacteria could be classified into a phylum and for those associated with ten liverworts, 14 phyla were identified. Of them, the phyla *Proteobacteria*, *Actinobacteria*, *Acidobacteria*, *Bacteroidetes*, *Armatimonadetes*, *Chloroflexi*, *Planctomycetes* and *Firmicutes* were detected in all ten liverworts, but bacteria assigned to *Fibrobacteres*, *Gemmatimonadetes*, *Chlamydiae*, TM6 and WCHB1-60 also appeared in some of the liverwort samples (Fig. 2a). Of the bacteria associated with mosses, seven phyla could be detected in all samples, including *Proteobacteria*, *Actinobacteria*, *Acidobacteria*, *Bacteroidetes*, *Armatimonadetes*, *Chlamydiae* and *Planctomycetes*. In addition, the five phyla *Fibrobacteres*, *Gemmatimonadetes*, *Firmicutes*, TM6 and WCHB1-60 were also detected in some of the mosses (Fig. 2b). Thus, the common phyla to all the bryophytes were *Proteobacteria*, *Actinobacteria*, *Acidobacteria*, *Bacteroidetes*, *Armatimonadetes* and *Planctomycetes*.

**Table 3:** Ratio of unclassified sequences at different taxonomic levels

	Relative abundance (%)				
Sample ID	Phylum	Class	Order	Family	Genus
JM1_T	0.00	0.12	4.53	8.59	26.25
JM2_T	0.00	0.08	6.57	15.25	30.06
JM28_T	0.00	0.32	0.83	8.39	18.11
JM30_T	0.00	0.07	2.37	4.73	31.05
GL3_T	0.04	1.71	7.16	9.85	33.26
GL7_T	0.01	0.10	1.18	5.70	26.63
GL9_T	0.12	0.24	2.39	6.50	18.54
GL10_T	0.31	0.93	6.35	9.28	18.59
GWL5_T	0.03	0.08	3.41	6.86	17.85
GWL7_T	0.13	0.19	7.84	12.03	28.38
JM3_X	0.00	0.14	3.97	5.10	23.47
JM5_X	0.00	0.01	1.25	4.17	37.94
JM25_X	0.00	0.23	3.17	6.54	27.37
JM29_X	0.00	0.02	12.66	14.07	62.47
TM2_X	0.00	0.01	15.41	15.52	61.79
GL1_X	0.01	0.07	10.58	12.85	54.00
GL4_X	0.02	0.90	5.10	7.10	26.50
GL5_X	0.01	0.22	6.71	7.08	16.99
GWL2_X	0.03	0.10	15.05	16.03	60.62
GWL9_X	0.00	0.00	7.71	8.86	45.01

Further analysis showed that *Proteobacteria* was the most predominant phylum inhabited in the nineteen samples, with the exception of the sample of *Hypnum revolutum* (JM5_X). In consideration of the dominant phyla associated with liverworts and mosses, there were no big differences among ten liverworts, and *Actinobacteria*, *Acidobacteria*, *Bacteroidetes* and *Armatimonadetes* were the subsequent dominant phyla associated with all of them. However, there were big differences among the bacteria associated with the ten mosses. For example, *Actinobacteria* was the most dominant phylum in *Hypnum revolutum* (JM5_X), and it was also the abundant phylum in the samples of *Racomitrium barbuloides* (GL1_X), *Pogonatum perichaetiale* (GL4_X), *Brachythecium rutabulum* (GWL9_X) and *Sanionia uncinata* (JM25_X). In contrast, it was infrequently detected in *Wijkia deflexifolia* (GL5_X), *Racomitrium sudeticum* (GWL2_X), *Pogonatum urnigerum* (JM3_X) and *Hypnum plumaeforme* (TM2_X). *Acidobacteria* was also abundant in seven of the mosses, but only a small quantity could be detected in GWL9_X, JM29_X and TM2_X.

**Fig. 3:**
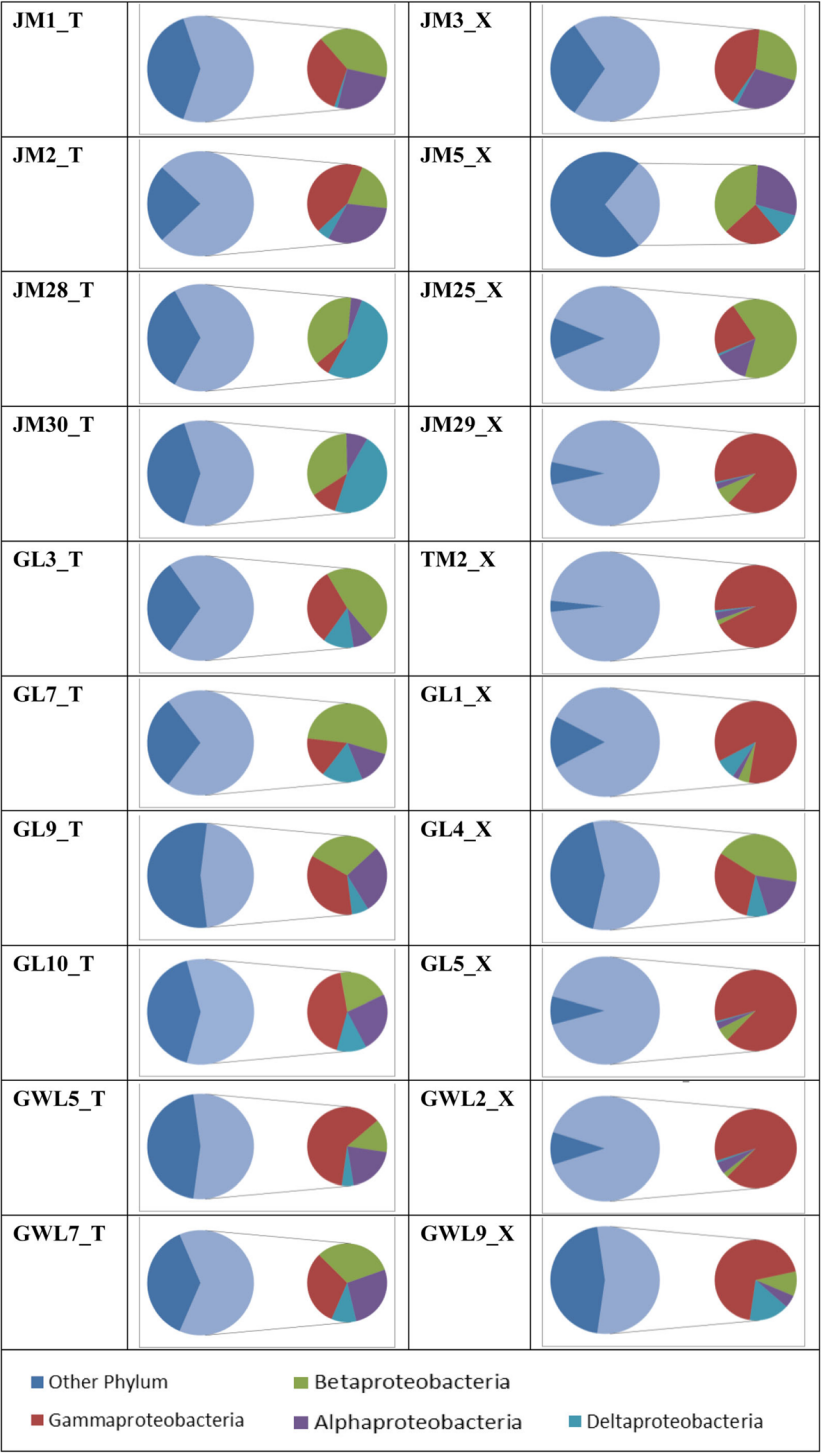
The distribution of all sequences from each bryophyte sample on the class level of phylum *Proteobacteria*

The phylum *Proteobacteria* that was detected in the libraries could be subdivided into four classes: *gamma*, *beta*, *alpha* and *delta*. Although the proportions of each class were different in different liverwort samples, all four classes were present in all but the abundance of *gamma* and *beta* classes were usually greater. In mosses, there were bigger differences among different samples. In four samples (JM29_X, TM2_X, GL5_X and GWL2_X), the *Proteobacteria* consisted of primarily the *gamma* class, while *beta*, *alpha* and *delta* classes were only present in a small proportion (Fig. 3). Thus, there were similar compositions of bacteria associated with the ten liverworts at both the phylum and class level of *Proteobacteria*, while large differences in bacterial composition existed among different moss species.

### Community structures of bacteria associated with the bryophytes at the genus level

**Fig. 4:**
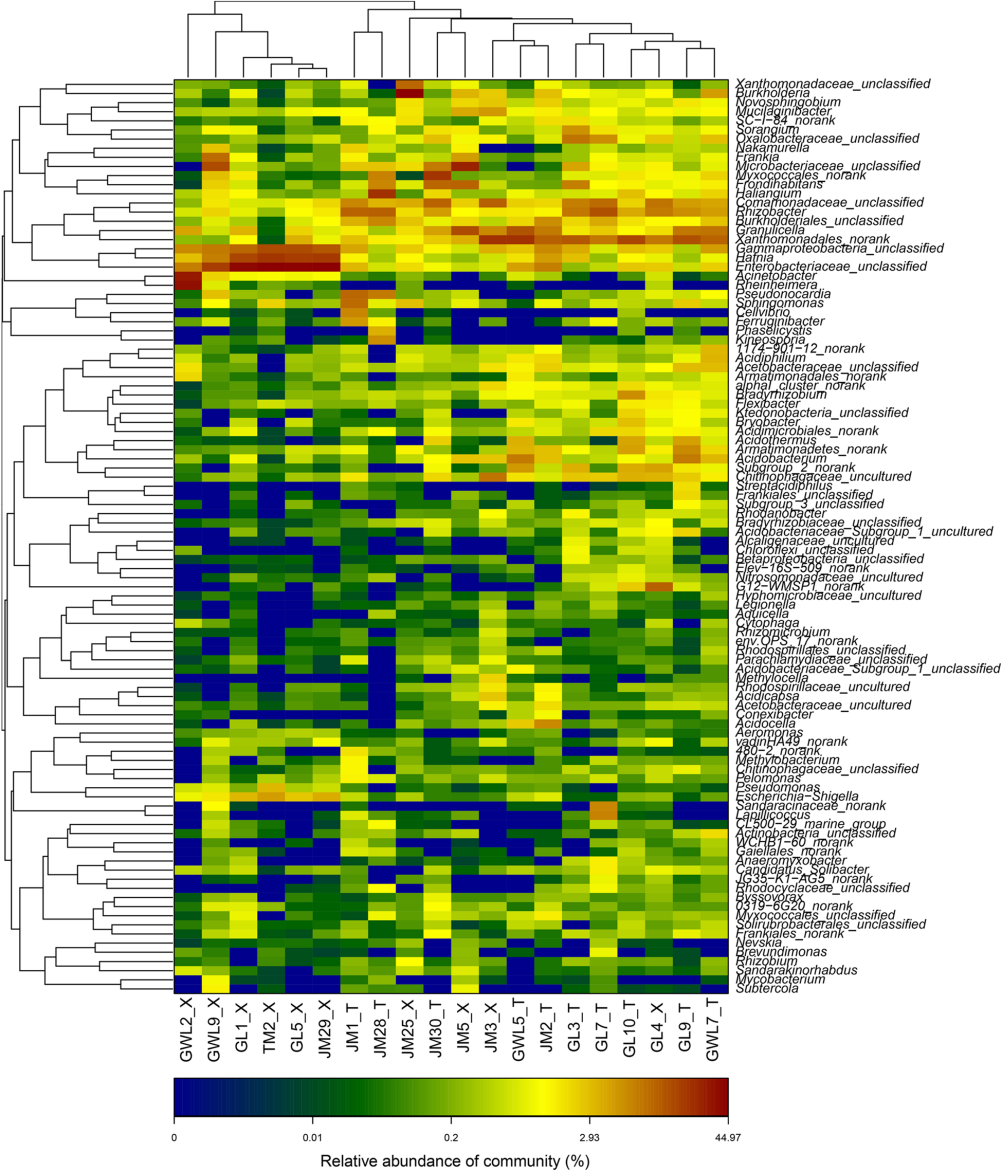
The Heatmap of bacterial composition in all bryophytes at genus level

According to the taxonomic analysis, a relatively large number of OTUs could not be assigned into any genus with a confidence level higher than 70%, suggesting that the presence of many unclassified sequences were detected in these bryophytes (Table 3). In total there were at least 176 genera of bacteria associated with the twenty bryophytes, and the first 100 abundant genera were reflected by the Heatmap diagram (Fig. 4). Of them, approximately 21 genera were shared by all the samples in a higher proportion, including 10 recognized genera such as *Burkholderia*, *Novosphingobium*, *Mucilaginibacter*, *Sorangium*, *Frankia*, *Frondihatitans*, *Haliangium*, *Rhizobacter*, *Granulicella* and *Hafnia*, and another 11 unclassified genera in family *Xanthomonadaceae*, *Oxalobacteraceae*, *Microbacteriaceae*, *Comamonadaceae* and *Enterobacteriaceae*, and some in the order *Myxococcales*, *Burkholderiales* and *Xanthomonadales*. But those in the genera of *Acidiphilum*, *Bradyrhizobium*, *Flexibacter*, *Bryobacter*, *Acidothermus*, *Acidobacterium,* and family of *Acetobacteraceae* and *Chitinophagaceae*, order of *Armatimonadales* and *Acidimicrobiales*, even some in the alphal_cluster of *Ktedonobacteria* and *Armatimonadetes*, were mainly associated with the liverworts. The bacterial community reflected by the heatmap that was based on the genus level showed that bacteria associated with six mosses (GWL2_X, GWL9_X, GL1_X, GL5_X, JM29_X and TM2_X) displayed a higher community similarity; whereas, the bacteria associated with the other four mosses exhibited a higher similarity with the liverworts.

### The bacterial community similarities among different bryophytes

**Fig. 5:**
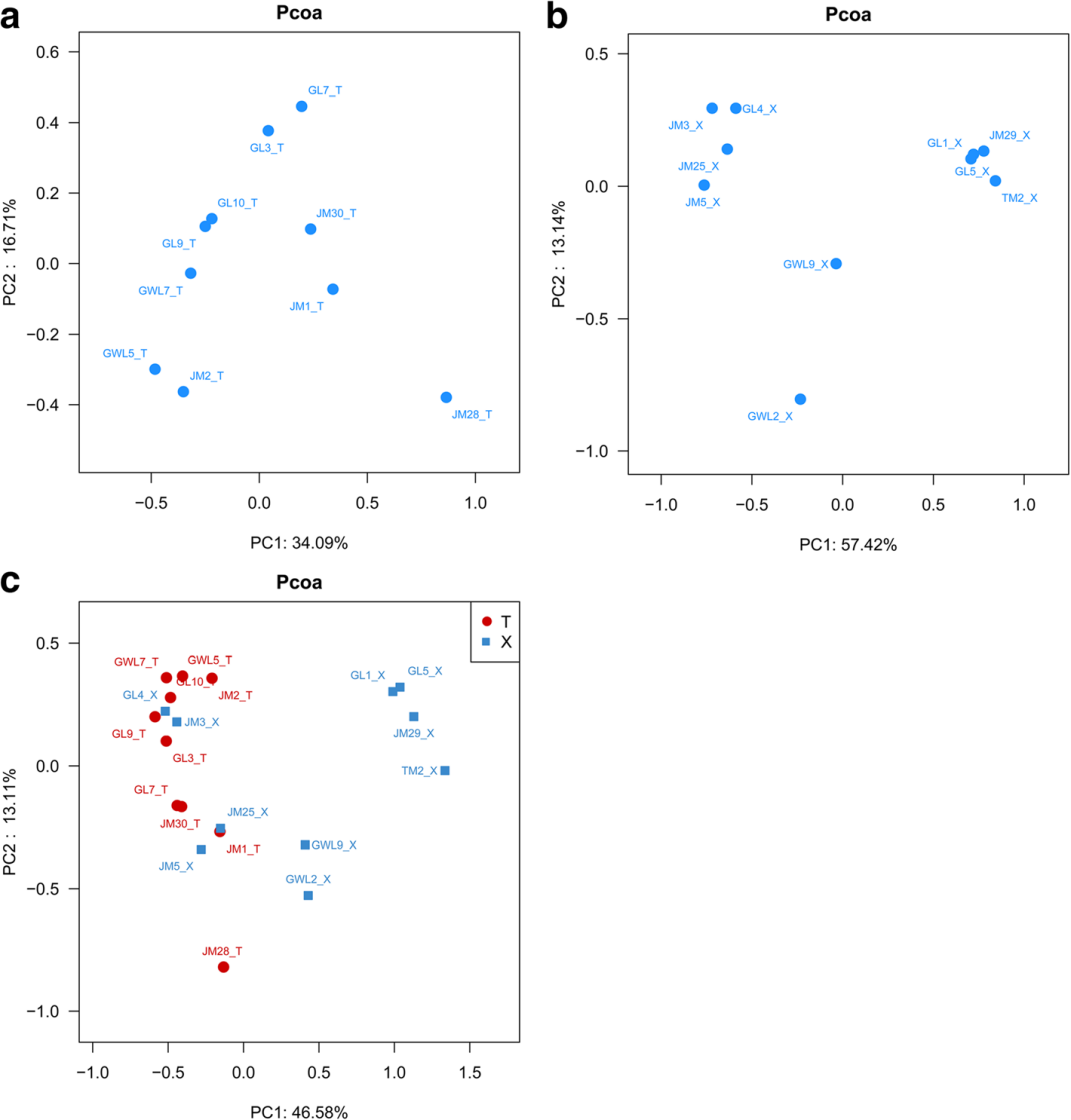
The PCoA analysis of the bacterial community in ten liverworts (**a**), ten mosses (**b**) and all twenty bryophytes (**c**)

In the PCoA diagram based on ten liverworts generated based on 50.80% variance (34.09% first axis and 16.71% second axis, Fig. 5a), although there was not obvious laws for the distribution of the samples in the plots, totally the bacterial community in four liverworts sampled from Galongla glacier (GL: GL7_T, GL3_T, GL9_T and GL10_T) and two from Gawalong glacier (GWL: GWL7_T and GWL5_T) displayed the relatively closer distances, respectively, while for another four liverworts collected from Zhamo roadside (JM), their associated bacterial community displayed a relatively bigger differences. For the PCoA analysis based on ten mosses, the bacterial community associated with them also exhibited that samples from the same site of JM (JM3_X, JM5_X, JM25_X), GL (GL1_X and GL5_X) and GWL (GWL2_X and GWL9_X) had a relatively closer distances with the exception of GL4_X, JM29_X and TM2_X based on 70.56% variance (57.42% first axis and 13.14% second axis, Fig. 5b). Interestingly, when the PCoA analysis was based on all twenty bryophytes, the bacteria associated with all the samples could be divided into two groups: (1) the bacteria community associated with the four specific mosses (JM3_X, JM5_X, JM25_X, GL4_X,) and all ten liverworts displayed a closer relationship. (2) bacteria associated with another six moss species (JM29_X, TM2_X, GL1_X, GL5_X, GWL2_X and GWL9_X) were more similar. The cumulative percentage variance of species was explained by 59.69% variance (Fig. 5c).

**Fig. 6:**
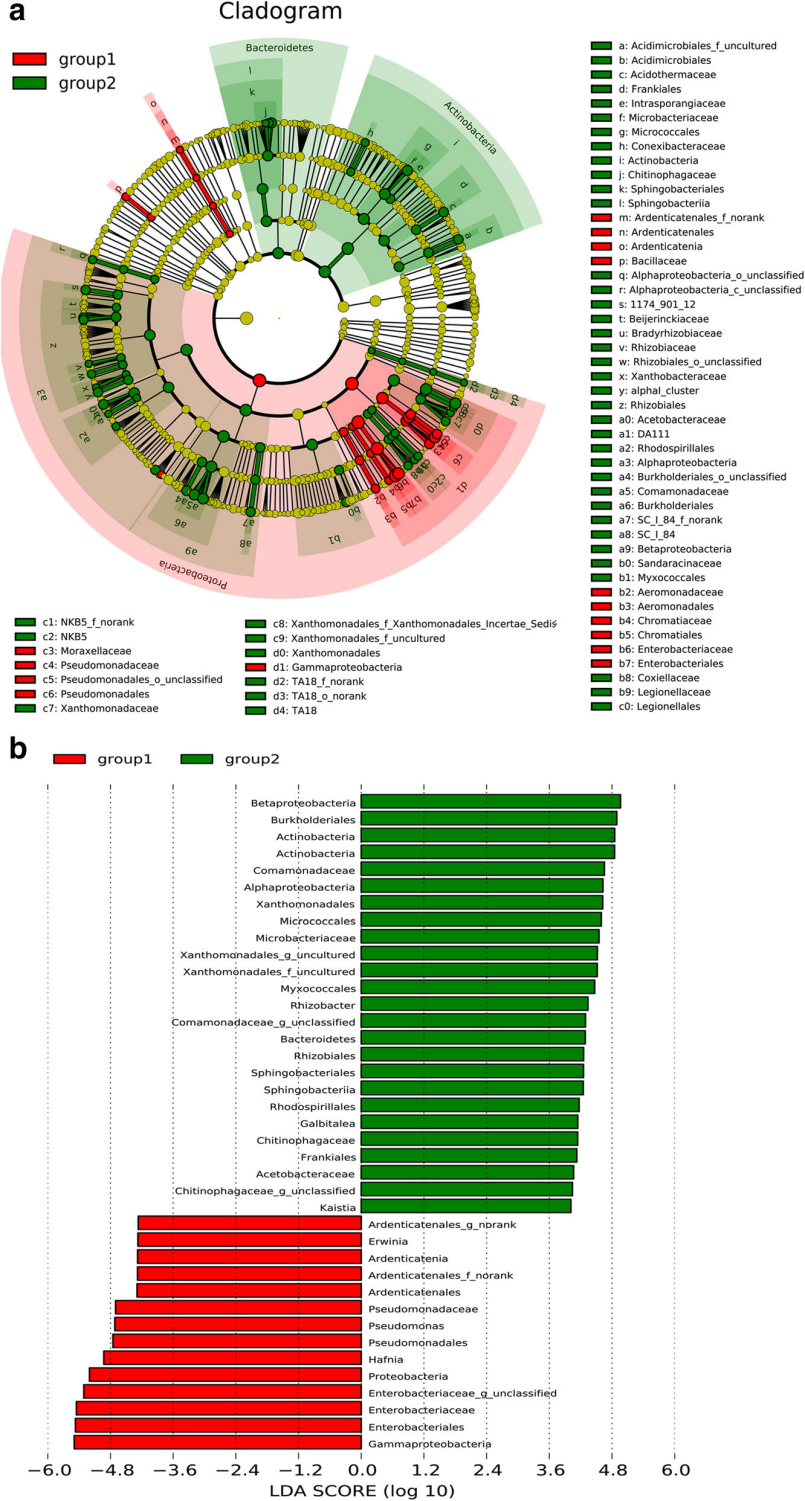
The LEfSe analysis of bacteria associated with part bryophytes of group 1 (samples JM29_X, TM2_X, GWL9_X, GWL2_X, GL1_X and GL5_X) and group 2 (samples JM3_X, JM5_X, JM25_X, GL4_X and ten liverworts). **a** Cladogram representing the taxonomic hierarchical structure of the identified phylotype biomarkers generated using LEfSe. Phylotype biomarkers were identified comparing samples from group 1 and group 2. Each filled circle represents one biomarker. Red, phylotypes statistically overrepresented under the condition of group 1; green, phylotypes overrepresented under the condition of group 2; yellow, phylotypes for which relative abundance is not significantly different between the two conditions. The diameter of each circle is proportional to the phylotype’s effect size, phylum is indicated in their names on the cladogram and the class, order, family, or genera are given in the key. **b** Identified phylotype biomarkers ranked by effect size in group1 and group 2. The phylotype biomarkers were identified as being significantly abundant when samples from group 1 and group 2 were compared and the alpha value was <0.05

The LEfSe analysis further identified the specific bacterial taxa that were differentially present or abundant in above two groups (six mosses made up group 1, and other 14 bryophytes were group 2). The results showed that bacteria in the order *Ardenticatenales* and family *Bacillaceae* were significant abundant in six of the mosses (Fig. 6a and b). In addition, four orders (*Enterobacteriales*, *Aeromonadales*, *Chromatiales* and *Pseudomonadales*) of bacteria in the *gamma* class of *Proteobacteria* were also significantly abundant in these 6 samples, and they were significantly differentiated from the other 14 bryophytes. In contrast, the bacteria that were more abundant in the 14 other samples compared to group 1 were those in: *Sphingobacteriales* and *Chitinophagaceae* of phylum *Bacteroidetes*; in *Conexibacteraceae*, *Microbacteriaceae*, *Acidothermaceae* and *Acidimicrobiales* of phylum *Actinobacteria*; in TA18 group; in *Xanthomonadales*, *Legionellales* and NKB5 group of the *gamma* class of *Proteobacteria*; in *Comamonadaceae*, SC_1_84, and the unclassified family of *Burkholderiales* in *betaproteobacteria*; and finally those in the order *Rhizobiales* and *Rhodospirillales* of *alphaproteobacteria*.

## Discussion

### Advantage of the combination of the nested-PCR system and illumina sequencing technique

The objective of this study was to evaluate the composition of the bacterial communities that are associated with different bryophytes using a high throughput-sequencing technique. Since the bacteria associated with the bryophytes either resided inside the plant or was attached to the surface, we used the total DNA that was extracted from the sampled bryophytes as the template. In order to control for the DNA of plant chloroplasts or mitochondria, a nested PCR technique was used in this study. Primers used in the first run were 799 F and 1492R, which have been used successfully to filter out chloroplast DNA and separate the bacterial 16S rRNA gene fragment from the mitochondria DNA of hosts [16]. The second run was based on the primer pairs of 926 F and 1392R and was used to further amplify the V6-V8 region of bacterial 16S rRNA gene. According to the data obtained in this survey, ≥ 99.9% of the reads obtained were bacterial 16S rRNA gene fragments. This confirmed that the nested PCR technique based on these two primer pairs could be successfully used for the high throughput sequencing of bacteria on plant hosts.

In line with previous investigations of bacterial communities and diversity inhabited in *Grimmia Montana* using 16S rDNA clone library techniques [13], the dominant bacterial phyla were *Proteobacteria*, *Actinobacteria*, *Firmicutes* and *Bacteriodetes*; while another bacterial-biota dynamics research made by Koua (2015) only detected two phyla of *Proteobacteria* and *Firmicutes* in the phyllosphere of the eight bryophytes from different ecosystems when using the PCR-DGGE technique based on the bacterial V3 region of 16S rRNA gene [15]. However, those in phyla *Acidobacteria*, *Armatimonadetes*, *Chlamydiae* and *Planctomycetes* were also detected in all the bryophytes within this study, and the bacteria affiliated to phyla *Firmicutes*, *Gemmatimonadetes*, TM6 and WCHB1-60 were also found in some of moss species, which showed that the nested-PCR combined with the Illumina-sequencing technique used here is a more sensitive and effective method for the identification of bacteria associated with plant hosts.

### The bacterial richness and diversity in different bryophytes

Here, we studied the bacteria associated with twenty bryophytes mainly derived from four places including four different niches, the roadside of Zhamo (JM) and Tongmai (TM) and the glaciers of Galongla (GL) and Gawalong (GWL). According to the OTU numbers, Chao estimators, Shannon diversity indexes and Simpson indexes, the richness and diversity of the bacteria associated with TM2_X were the lowest on the scale. This may be due to the altitude (2000 m) of the sampling site of TM at Tongmai, which was far lower than those collected from the higher sampling locations (4100 ~ 4337 m). Equally, there was no relationship between the bacterial richness or diversity in samples collected from all other sampling sites, JM, GL or GWL, which are at similar altitudes. Thus, higher altitudes might be helpful to stimulate bacteria inhabitance in or on hosts as a way to help them adapt to extreme environments.

### Comparison of bacteria community between those associated with common higher plants and bryophytes

Sequencing taxonomy analysis showed that the bacteria that inhabit bryophytes are primarily members of eight to twelve specific phyla. Of them, *Proteobacteria*, *Actinobacteria*, *Acidobacteria* and *Bacteriodetes* were the dominant phyla in all samples. Consistent with other reports, the members of phylum *Proteobacteria* were also detected as the most dominant bacteria in many plant hosts, and the proportion of bacteria in other phyla were differentially varied across the different hosts [15, 16, 25–27]. Compared with the dominant bacteria associated with other common higher plants, *Acidobacteria* and *Actinobacteria* were still in high proportions, and those in *Armatimonadetes*, *Planctomycetes*, *Chloroflexi*, *Gemmatimonadetes*, *Firmicutes* and group TM6 and WCHB1-60, which were rarely detected in higher plants, were found in the majority of the bryophytes examined in this survey.

*Acidobacteria* is the second most dominant phylum inhabited in soil, sediments or benthonic species and its persistence could be based on the general mechanism of trace gas oxidation [28]. In this study, we detected it in nearly all the bryophytes at varied levels (0.15 ~ 30.54%). Although we don’t understand its role on the bryophytes, the bryophytes appeared to be good hosts and could provide suitable growth conditions for many *Acidobacteria*.

The bacterium of *Armatimonadetes* was originally described solely on the basis of environmental 16S rRNA gene clone sequences, and a bacterial strain *Armatimonas rosea* of this phylum was first isolated from an aquatic plant in Japan in 2011 [29]. The report of the first *Armatimonadetes* genome from the thermophile *Chthonomonas calidirosea* T49 (T) showed that it could act as a saccharide scavenger in a geothermal steam-affected soil environment. Its predicted genes encode for carbohydrate transporters and carbohydrate-metabolizing enzymes, which would help the plant hosts utilize a wide range of carbohydrates [30]. Small amounts of *Armatimonadetes* bacteria detected in all bryophytes showed that they are important to the micro-ecosystem of hosts by helping them utilize a wide range of carbohydrates to prompt their growth. Likewise *Planctomycetes*, which are aquatic bacteria that often inhabit brackish and fresh water, were identified by a stable-isotope probing technique to degrade complex heteropolysaccharides in soil [31]. In this survey, we demonstrated that they also inhabit bryophytes, which are a type of lower group derived from water and depend on nutrient resources and rainwater.

*Chloroflexi* is a phylum of bacteria containing isolates with a diversity of phenotypes, including members that are aerobic thermophiles and use oxygen and grow well in high temperatures; anoxygenic phototrophs that use light for photosynthesis; and anaerobic halorespirers that act as energy sources. Barton and colleagues (2014) reported that the microbial diversity in a Venezuelan orthoquartzite cave was dominated by the *Chloroflexi* (Class *Ktedonobacterales*) and postulated that the poor buffering capacity of quartzite or the low pH of the environment selected for this unusual community structure [32]. Interestingly, some of these were also detected in the majority of bryophytes and could be related to the extreme niches of the bryophyte locations.

*Gemmatimonadetes* have been found in a variety of arid soils, such as grassland, prairie, and pasture soil, as well as eutrophic lake sediments and alpine soils. The phylum *Gemmatimonadetes* diverged in early microbial evolution at least 3 billion years ago [33]. As the living fossil of plants, bryophytes might be good hosts for their growth and production, and thus, it was not particularly surprising that this phylum was also detected in this study.

In addition to those listed above, bacteria within the phylum TM6 are those chiefly found in the biofilm of sink drains and are considered to be the primary low abundance members in a wide range of habitats, as identified by culture-independent rRNA surveys. Several genomes of these bacteria were analyzed by two independent research groups, McLean (2013) and Yoeh (2016). Both studies found that these bacteria are small and lack complete biosynthetic pathways for various essential cellular building blocks including amino acids, lipids, and nucleotides. Other features identified in the TM6 genomes included a degenerated cell envelope, the expression of ATP/ADP translocases for parasitizing host ATP pools, and protein motifs that facilitate eukaryotic host interactions [34, 35]. Another uncultured phylum, the WCHB1-60, was detected in pond samples, although the possible functions of these phyla in the liverworts and mosses are not clear and should be considered in future research.

Thus, there were many differences between the bacterial communities associated with bryophytes compared to those in the common higher plants. The bacteria associated with bryophytes included those adapted to aquatic, drought and even anaerobic environments, which is consistent with the bryophyte transition from aquatic to terrestrial conditions. In addition, there were a small proportion of sequences assigned to unclassified phylum and many of them assigned to unclassified genera, suggesting that there are large amounts of new symbiotic bacteria associated with bryophytes.

### The factors involved in the bacterial community similarities among different bryophytes

Combined analyses of the bacteria associated with ten liverworts via Heatmap and PCoA patterns (Fig. 4a) showed that samples from each same sampling site of Gawalong glacier (GWL), Galongla glacier (GL) and Zhamo roadside (JM) generally displayed more similar bacterial communities, although the bacterial community associated with the liverworts from JM displayed more scattered distribution. Thus, we inferred that the bacterial community similarity was primarily predictive of the sampling site itself. Each sampling site represented a specific environmental niche; therefore, the niche was considered to be a determining factor of bacterial community in the liverworts to some extent. It was deeply confirmed by the relatively closer distribution of samples from two different glacier regions, GL and GWL, which also showed that the similar bacterial community compositions inhabited in/on the liverworts from similar niches might be more similar. In addition, although the samples JM30_T and GL10_T were both *Jungermannia parviperiantha* of order *Jungermanniales*, and collected from Zhaomo roadside and Galongla glacier respectively, their bacterial compositions were shown in a lower similarity by the relatively far distance in the first axis of PCoA plot (Fig. 4a). Thus, it further suggested that the niches were likely the most important factor to determine which bacteria could inhabit in/on liverworts in this survey.

Furthermore, the bacteria associated with ten mosses were divided into three groups (Fig. 4b) and totally displayed that the same sampling site derived samples nearly had the higher bacterial community similarity, but with the exception of GL4_X, JM29_X and TM2_X. The exception of the bacteria associated with these three mosses suggested that there must be other factors influenced on the bacterial composition. Further analysis the phylogenetic status of sample GL4_X and found that it was a species of genus *Pogonatum*, family *Polytrichaceae* and belonged to the same genus and with very close relationship to sample JM3_X (shown in Table 1); while JM29_X were the species *Racomitrium himalayanum*, and belonged to the same genus with samples GL1_X and GL5_X., which meant JM29_X was phylogenetically closely related to GL1_X and GL5_X. According to the PCoA pattern (Fig. 4b), the bacteria associated with GL4_X showed the higher similarity with those inhabited in/on JM3_X, JM5_X and JM25_X, while bacteria inhabited in /on JM29_X showed the higher similarity with those in GL1_X and GL5_X, which demonstrated that the phylogenetic status of the hosts might be another important factor (besides sampling site) that could influence the bacterial community in mosses. For TM2_X, although it was identified as the same genus of *Hypum* with sample JM5_X, the reason why they were not clustered together probably related to the characters of genus *Hypum* in order *Hypnales*. As reporting on the phylogenetic research of different mosses by the molecular methods [36], order *Hypnales* formed a large clade with the inclusion of exemplars of pleurocarpous mosses, some families such as *Brachytheciaceae, Amblystegiaceae* and *Sematophyllaceae* appeared as monophyletic entities, whereas *Hypnaceae,* as another family of *Hypnales,* was polyphyletic and the genus *Hypnum* was also placed in several distinct clades. Thus, it might be the reason why the bacteria in sample TM2_X and JM5_X were not clustered together.

However, when the PCoA profile was made based on all 20 bryophytes (Fig. 4c), the bacterial communities of four mosses (JM3_X, JM5_X, JM25_X, GL4_X,) and ten liverworts were clustered together, showing that their associated bacterial communities were more similar to each other than to the other six mosses. Further analysis of the genetic characteristics of these bryophytes also confirmed that the phylogenetic relationships of these bryophytes might play an important role in determining which bacteria inhabitance with the hosts. For example, ten samples of the liverworts belonged to the order of *Jungermanniales* or *Marchantiales*, respectively, while the ten mosses were from three different orders, *Hypnales*, *Polytrichales* and *Grimmiales*. According to the phylogeny status of bryophytes previously described, bryophytes represent the first green plants to colonize the terrestrial environment. This plant type includes liverworts, hornworts and mosses. Some analyses that have combined morphological characters with rRNA sequences and that of the chloroplast gene rbcL [37, 38] have generally supported a liverworts-basal topology (LBT) and placed either mosses or hornworts as sister to vascular plants. Other reports based on extensive cladistics analysis of morphological, ultrastructural data and mitochondrial small-subunit (mtSSU) rDNA [39, 40] yielded a monophyletic moss-liverwort clade, with hornworts sister to all land plants (hornworts-basal topology, HBT). Thus, whether the phylogeny of bryophytes was supported by LBT or HBT hypothesis, liverworts had little differentiation on organs such as stem or leaf, and were lower than mosses in the taxonomic status. The four mosses that were clustered together with liverworts, the JM3_X, GL4_X JM5_X and JM25_X, belonged to the families *Polytrichaceae* of order *polytrichales*, and *Hypnaceae*, *Amblystegiacae* of order *Hypnales*, respectively, whereas another three mosses GL1_X, GL5_X and JM29_X belonged to *Grimmiaceae* of order *Grimmiales*, and GWL9_X, GWL2_X and TM2_X belonged to the family of *Brachytheciaceae*, *Sematophyllaceae* and *Hypnaceae* of *Hypnales*, respectively (Shown in Table 1). According to the molecular phylogenetics and ordinal relationships based on analyses of a large-scale data set of 600 rbcL sequences of mosses [36], *Polytrichaceae* is the representative of *Polytrichales* and formed the basal placements clade in these mosses, while the order *Grimmmiales* and *Hypnales* were considered as the sister clade in a relatively higher phylogenetic status of the mosses [41]. In addition, order *Hypnales* formed a large clade, some involved families such as *Amblystegiaceae*, *Sematophyllaceae* and *Brachytheciaceae* appeared as monophyletic entities [36], but *Hypnaceae* was polyphyletic, and one of its genus *Hypnum* was also placed in different clades. Thus, the reason why the four mosses of JM5_X, JM25_X, JM3_X and GL4_X had similar bacterial communities with ten liverworts was likely due to the phylogenetic status of *Polytrichaceae* (JM3_X and GL4_X) were closer to the liverworts. In addition, although *Amblystegiaceae* (JM25_X) and some species in genus of *Hypnum* of *Hypnaceae* (JM5_X) were assigned to a higher phylogenetic status of order *Hypnales*, their differentiate extent might be more close to *Polytrichales* and liverworts. While the other six mosses, in which three of them belonged to *Grimmiaceae* in order *Grimmiales* (GL1_X, GL5_X and JM29_X), one *Brachytheciaceae* (GWL9_X) and one *Sematophyllaceae* species in *Hypnales* (GWL2_X) and another *Hypnum* species (TM2_X), were grouped together because it might be related to they (family *Grimmiaceae*, *Brachytheciaceae*, *Sematophyllaceae* and some *Hypnum* species) were in a higher and closer phylogenetic position in bryopsida and further away from the liverworts.

## Conclusions

In this survey, the bacterial community similarity among different bryophytes seemed related to their phylogenetic position and niches of hosts inhabited. For the hosts with the closer phylogenetic status, the bacterium associated with them was more similar. For those with a very similar phylogenetic status like ten liverworts and some mosses samples, their bacterial community was mainly related to the niches of samples. This hypothesis was basically consistent with reports by Dynesius [42], who concluded that tolerance to ash treatment of different bryophytes was more related to phylogeny than ecology when examining the responses of bryophytes to wood-ash recycling. Certainly, further studies with more bryophytes genotypes will still be needed to determine if this is consistent for all bryophytes or limited to those studied here. In addition, further isolation and identification of more potential useful bacterial resources (especially abundant in different groups) and enhancing the work on their possible biological function in the hosts would be very necessary to make a good understanding on the interaction mechanism between bryophytes and microbes.

## References

[1] Hallmann J, Quadt-Hallmann A, Mahaffee WF, Kloepper JW (1997). Bacterial endophytes in agricultural crops. Can J Microbiol.

[2] Strobel G, Daisy B, Castillo U, Harper J (2004). Natural products from endophytic microorganisms. J Nat Prod.

[3] Mastretta C, Barac T, Vangronsveld J, Newman L, Taghavi S, Van der Lelie D (2006). Endophytic bacteria and their potential application to improve the phytoremediation of contaminated environments. Biotech Gen Eng Rev.

[4] Afzal M, Khan QM, Sessitsch A (2014). Endophytic bacteria: prospects and applications for the phytoremediation of organic pollutants. Chemosphere.

[5] Raymond JA (2016). Dependence on epiphytic bacteria for freezing protection in an Antarctic moss, *Bryum argenteum*. Environ Microbiol Rep.

[6] Brock T, Bregman R (1989). Periodicity in growth, productivity, nutrient content and decomposition of *Sphagnum recurvum* var. *mucronatum* in a fen woodland. Oecologia.

[7] Opelt K, Berg G (2004). Diversity and antagonistic potential of bacteria associated with bryophytes from nutrient-poor habitats of the Baltic Sea coast. Appl Environ Microbiol.

[8] Opelt K, Berg C, Schoenmann S, Eberl L, Berg G (2007). High specificity but contrasting biodiversity of *Sphagnum*-associated bacterial and plant communities in bog ecosystems independent of the geographical region. ISME J.

[9] Opelt K, Chobot V, Hadacek F, Schoenmann S, Eberl L, Berg G (2007). Investigations of the structure and function of bacterial communities associated with Sphagnum mosses. Environ Microbiol.

[10] Nickrent DL, Parkinson CL, Palmer JD, Duff RJ (2000). Multigene phylogeny of land plants with special reference to bryophytes and the earliest land plants. Mol Biol Evol.

[11] Ye J, Hao ZQ, Yu DY, Yan HB, Feng DQ (2004). Research advances in bryophyte ecological function. Chin J Appl Ecol.

[12] Turetsky MR (2003). The role of bryophytes in carbon and nitrogen cycling. Bryologist.

[13] Liu XL, Liu SL, Liu M, Kong BH, Liu L, Li YH (2014). A primary assessment of the endophytic bacterial community in a xerophilous moss (*Grimmia montana*) using molecular method and cultivated isolates. Brazn J Microbiol.

[14] Opelt K, Berg C, Berg G (2007). The bryophyte genus *Sphagnum* is a reservoir for powerful and extraordinary antagonists and potentially facultative human pathogens. FEMS Microbiol Ecol.

[15] Koua FHM, Kimbara K, Tani A (2015). Bacterial-biota dynamics of eight bryophyte species from different ecosystems. Saudi J Biol Sci.

[16] Chelius M, Triplett E (2001). The diversity of archaea and bacteria in association with the roots of *Zea mays* L. Microb Ecol.

[17] Magoc T, Salzberg SL (2011). FLASH: fast length adjustment of short reads to improve genome assemblies. Bioinformatics.

[18] Caporaso JG, Kuczynski J, Stombaugh J, Bittinger K, Bushman FD, Costello EK, Fierer N, Pena AG, Goodrich JK, Gordon JI (2010). QIIME allows analysis of high-throughput community sequencing data. Nat Methods.

[19] Edgar RC (2013). UPARSE: highly accurate OTU sequences from microbial amplicon reads. Nat Methods.

[20] Edgar RC, Haas BJ, Clemente JC, Quince C, Knight R (2011). UCHIME improves sensitivity and speed of chimera detection. Bioinformatics.

[21] Schloss PD, Westcott SL, Ryabin T, Hall JR, Hartmann M, Hollister EB, Lesniewski RA, Oakley BB, Parks DH, Robinson CJ (2009). Introducing mothur: Open-source, platform-independent, community-supported software for describing and comparing microbial communities. Appl Environ Microbiol.

[22] Chao A (1984). Nonparameteric-estimation of the number of classes in a population. Scand J Stat.

[23] Cole JR, Wang Q, Cardenas E, Fish J, Chai B, Farris RJ, Kulam-Syed-Mohideen AS, McGarrell DM, Marsh T, Garrity GM (2009). The ribosomal database project: improved alignments and new tools for rRNA analysis. Nucleic Acids Res.

[24] Quast C, Pruesse E, Yilmaz P, Gerken J, Schweer T, Yarza P, Peplies J, Gloeckner FO (2013). The SILVA ribosomal RNA gene database project: improved data processing and web-based tools. Nucleic Acids Res.

[25] Li YH, Liu QF, Liu Y, Zhu JN, Zhang QA (2011). Endophytic bacterial diversity in roots of *Typha angustifolia* L. in the constructed Beijing Cuihu Wetland (China). Res Microbiol.

[26] Faria DC, Dias AC, Melo IS, de Carvalho Costa FE (2013). Endophytic bacteria isolated from orchid and their potential to promote plant growth. World J Microbiol Biotechnol.

[27] Koiv V (2015). Microbial population dynamics in response to *Pectobacterium atrosepticum* infection in potato tubers. Sci Rep.

[28] O’Connor-Sanchez A, Rivera-Dominguez AJ, De los Santos-Briones C, Lopez-Aguiar LK, Pena-Ramirez YJ, Prieto-Davo A (2014). Acidobacteria appear to dominate the microbiome of two sympatric Caribbean Sponges and one Zoanthid. Biolog Res.

[29] Tamaki H, Tanaka Y, Matsuzawa H, Muramatsu M, Meng X-Y, Hanada S, Mori K, Kamagata Y (2011). *Armatimonas rosea* gen. nov., sp nov., of a novel bacterial phylum, Armatimonadetes phyl. nov., formally called the candidate phylum OP10. Int J Syst Evol Microbiol.

[30] Lee KCY, Morgan XC, Dunfield PF, Tamas I, McDonald IR, Stott MB (2014). Genomic analysis of *Chthonomonas calidirosea*, the first sequenced isolate of the phylum Armatimonadetes. ISME J.

[31] Wang XQ, Sharp CE, Jones GM, Grasby SE, Brady AL, Dunfield PF (2015). Stable-isotope probing identifies uncultured Planctomycetes as primary degraders of a complex heteropolysaccharide in soil. Appl Environ Microbiol.

[32] Barton HA, Giarrizzo JG, Suarez P, Robertson CE, Broering MJ, Banks ED, Vaishampayan PA, Venkateswaran K (2014). Microbial diversity in a Venezuelan orthoquartzite cave is dominated by the Chloroflexi (Class Ktedonobacterales) and Thaumarchaeota Group I.1c. Front Microbiol.

[33] Takaichi S, Maoka T, Takasaki K, Hanada S (2010). Carotenoids of *Gemmatimonas aurantiaca* (Gemmatimonadetes): identification of a novel carotenoid, deoxyoscillol 2-rhamnoside, and proposed biosynthetic pathway of oscillol 2,2’-dirhamnoside. Microbiology-SGM.

[34] McLean JS, Lombardo MJ, Badger JH, Edlund A, Novotny M, Yee-Greenbaum J, Vyahhi N, Hall AP, Yang Y, Dupont CL (2013). Candidate phylum TM6 genome recovered from a hospital sink biofilm provides genomic insights into this uncultivated phylum. Proc Nat Acad Sci USA.

[35] Yeoh YK, Sekiguchi Y, Parks DH, Hugenholtz P (2016). Comparative genomics of candidate phylum TM6 suggests that parasitism is widespread and ancestral in this lineage. Mol Biol Evol.

[36] Tsubota H, Luna ED, Gonzalez D, Ignatov MS, Deguchi H (2004). Molecular phylogenetics and ordinal relationships based on analyses of a large-scale data set of 600 rbcL sequences of mosses. Hikobia.

[37] Shanker A, Sharma V, Daniell H (2011). Phylogenomic evidence of bryophytes’ monophyly using complete and incomplete data sets from chloroplast proteomes. J Plant Biochem Biotech.

[38] Zhang L, Zhang A, Chen J, Liu Y (2012). Phylogenetic relationship in some bryophytes based on rbcL-a gene of their chloroplast. Biotechnology.

[39] Beckert S, Steinhauser S, Muhle H, Knoop V (1999). A molecular phylogeny of bryophytes based on nucleotide sequences of the mitochondrial *nad5* gene. Plant Syst Evol.

[40] Nishiyama T, Kato M (1999). Molecular phylogenetic analysis among bryophytes and tracheophytes based on combined data of plastid coded genes and the 18S rRNA gene. Mol Biol Evol.

[41] Wahrmund U, Rein T, Muller KF, Groth-Malonek M, Knoop V (2009). Fifty mosses on five trees: comparing phylogenetic information in three types of non-coding mitochondrial DNA and two chloroplast loci. Plant Syst Evol.

[42] Dynesius M. Responses of bryophytes to wood-ash recycling are related to their phylogeny and pH ecology. Per Plant Ecol Evol Syst. 2012;14(1):21–31.

